# Unveiling the functional roles of patient‐derived tumour organoids in assessing the tumour microenvironment and immunotherapy

**DOI:** 10.1002/ctm2.1802

**Published:** 2024-09-08

**Authors:** Di Chen, Lixia Xu, Mengjuan Xuan, Qingfei Chu, Chen Xue

**Affiliations:** ^1^ Department of Neurosurgery The First Affiliated Hospital of Zhengzhou University Zhengzhou Henan China; ^2^ Department of Infectious Diseases The First Affiliated Hospital of Zhengzhou University Zhengzhou Henan China; ^3^ Department of State Key Laboratory for Diagnosis and Treatment of Infectious Diseases, National Clinical Research Center for Infectious Diseases, National Medical Center for Infectious Diseases, Collaborative Innovation Center for Diagnosis and Treatment of Infectious Diseases The First Affiliated Hospital Zhejiang University School of Medicine Hangzhou China

**Keywords:** cancer, immunotherapy, organoids, tumour microenvironment

## Abstract

Recent studies have established the pivotal roles of patient‐derived tumour organoids (PDTOs), innovative three‐dimensional (3D) culture systems, in various biological and medical applications. PDTOs, as promising tools, have been established and extensively used for drug screening, prediction of immune response and assessment of immunotherapeutic effectiveness in various cancer types, including glioma, ovarian cancer and so on. The overarching goal is to facilitate the translation of new therapeutic modalities to guide personalised immunotherapy. Notably, there has been a recent surge of interest in the co‐culture of PDTOs with immune cells to investigate the dynamic interactions between tumour cells and immune microenvironment. A comprehensive and in‐depth investigation is necessary to enhance our understanding of PDTOs as promising testing platforms for cancer immunotherapy. This review mainly focuses on the latest updates on the applications and challenges of PDTO‐based methods in anti‐cancer immune responses. We strive to provide a comprehensive understanding of the potential and prospects of PDTO‐based technologies as next‐generation strategies for advancing immunotherapy approaches.

## Graphical abstract

Using 3D platforms to monitor the cancer immune response

Organoids displayed preferable potential for cancer immunotherapy

Novel technologies to generate immune‐tumour organoids

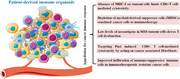

## 1 BACKGROUND

With the alarming increase in cancer incidence and mortality rates, cancer has become a significant global challenge, posing a severe threat to public health and economic prosperity. In 2022, the United States was projected to experience almost 610 000 cancer‐related deaths and the diagnosis of 1.91 million new cancer cases.^1^ Although various therapeutic strategies, including surgical resection, radiotherapy and chemotherapy, have made substantial progress in cancer management in patients,^2^ they are accompanied by certain drawbacks, such as adverse side effects, long‐term complications and the development of resistance to treatment.^3^ Future research efforts must address these limitations by pioneering innovative approaches to cancer therapy.

Immunotherapy, an encouraging therapeutic approach, has shown the potential to effectively enhance the immune system's ability to recognise and eliminate tumour cells. By improving the body's natural immune response, immunotherapy tackles the mechanisms that enable cancer cells to evade immune surveillance.^4^, ^5^ Current cancer immunotherapies methods include immune checkpoint inhibitors (ICIs), adoptive cell therapies (ACTs), cancer vaccination and oncolytic viruses.^6^ The United States Food and Drug Administration has approved several immunotherapeutic agents for cancer treatment, such as cytotoxic T‐lymphocyte‐associated protein 4 (CTLA‐4) antibodies, programmed cell death 1 (PD‐1) inhibitors and programmed cell death 1 ligand 1 (PD‐L1) inhibitors.^7^, ^8^ However, not all patients respond positively to these treatments due to tumour heterogeneity and limited immunogenicity. Additionally, some patients who initially respond to immunotherapy may develop resistance. Thus, it is important to develop models to accurately predict the efficacy of immunotherapy

In numerous studies, conventional technologies (Table 1), such as in vivo mouse models, have demonstrated their potential in assessing the therapeutic response to cancer.^9^ These successful in vivo preclinical models could transplant patient‐derived tumour fragments or cells into immunodeficient organisms (e.g., mice) subcutaneously or orthotopically, consequently preserving the complex interactions among microenvironmental components. Although frequently used to explore the tumour biology and drug responses, the animal models have some shortcomings due to the high cost, ethical concerns, low and/or delayed engraftment rates and discrepancy of pharmacodynamics and pharmacokinetics between mice and humans.^10^ Especially, the differences in the immune systems in mouse models making them inadequate for accurately replicating the complexity of the human immune microenvironment in cancer and resulting in unsatisfactory predictions of the efficacy of immunotherapy.^11^ Because of cost effectiveness, simplicity and long passage time, traditional two‐dimensional (2D) cell culture systems provide a stable model for various in vitro experiments. Furthermore, cancer cells from patients could be easily modified for mechanistic studies, drug testing and molecular biology research. However, cancer cell lines do not faithfully represent the genetic diversity and intricacies within tumours in human body.^12^ In the past decade, using liquid biopsy for interpretation of cancer‐related signals has generated great interest. Especially, these technologies based on biological fluids show great potential for analysing cancer immunity and predicting immunotherapy. The content changes of circulating components, such as immune cells, cytokines and chemokines, in blood could indicate the dynamic status of immunity, providing clues about the immune activation or suppression in tumour microenvironment (TME).^13^, ^14^ Nevertheless, some liquid biopsy strategies lack the sensitivity and specificity required to early detect of cancer treatment response.^15^ To address these limitations, patient‐derived tumour organoids (PDTOs) have emerged as advance culture tools to streamline the preclinical evaluation of therapeutic response with simplicity and high reliability. Three‐dimensional (3D) organoids are constructed through co‐culturing pluripotent stem cells (PSCs) and mature cells in culture medium supplement with specific growth factors and signalling inhibitors.^16^ Traditional tumour organoids are composed solely of malignant cells, allowing for the exploration of tumour cell‐specific biological behaviours. They exhibit complex tissue architecture and sufficiently capture the histopathological heterogeneity, genotypes and phenotypes of original tumours, making them as attractive tumour‐specific models (Table 2). Even though, these conventional cancer organoids lack the stromal components, especially immune cells. The restricted incorporation of immune or stromal cells limits traditional organoids to accurately mimic TME and responses to treatment.^17^, ^18^, ^19^ More complex organoid systems that integrate both tumour cells and immune components is of paramount importance.

**TABLE 1:** The characteristics of several tumour systems in cancer research.

Models	Advantage	Disadvantage
2D cell lines	Cost effectiveness, simplicity and long‐passaged	Cannot faithfully represent the genetic diversity and intricacies within tumours in human body
Animal models	Preserving microenvironmental components Frequently used to explore the in vivo tumour biology and drug responses	Cannot faithfully replicate the complexity of human immune systems High cost, ethical concerns, low and/or delayed engraftment rates. Discrepancy of pharmacodynamics and pharmacokinetics between mice and humans
Liquid biopsy	The content changes of circulating components, such as immune cells, cytokines and chemokines, in blood could indicate the dynamic status of immunity.	Lacking the sensitivity and specificity required to early detect of cancer treatment response
Traditional organoids	Exhibiting complex tissue architecture, and sufficiently capturing the histopathological heterogeneity, genotypes and phenotypes of original tumours	Cannot robustly reproduce the intricacies of immunological processes because of limited infiltration of immune populations
Immune organoids	By introducing native stromal components, immune cells or exogenous immune elements, immune organoids can be utilised to truly model tumour microenvironment in patients.	Translated into clinical practice holds some questions, such as experimental standardisation, long‐term cultivation and accurately maintaining cellular complexity

**TABLE 2:** The definition and characteristics about organoids, organotypic cell clusters, micro‐organospheres and spheroids.

Models	Definition	Characteristics
Organoids	Constructed through co‐culturing pluripotent stem cells and mature cells in culture medium supplement with specific growth factors and signalling inhibitors	Exhibiting complex tissue architecture, and sufficiently capture the histopathological heterogeneity, genotypes and phenotypes of original tumours
Organotypic cell clusters	Formed by the aggregation of cells in conditioned culture	Exhibiting some specific tissue‐like structures and functions, but typically less organised and complex than traditional organoids
Micro‐organospheres	Small spherical structures formed by the aggregation of cells in suspension culture	Capturing multiple types of cells from the original tissues, including stromal cells and lymphocytes, but typically smaller and less complex than traditional organoids
Spheroids	Formed by the self‐assembly of cells in suspension or on non‐adherent surfaces	Mimicking the cellular compositions and dynamic cell–cell communication observed in host tumours, but lack the complex tissue architecture found in organoids

Nowadays, by introducing native stromal components, immune cells or exogenous immune elements, organoids can also be successfully utilised to truly model TME in patients, named as immune organoids. For example, the melanoma patient‐derived immune organoids, embedded in collagen gel or Matrigel, contain multiple immune cells, including CD4^+^ T cells, CD8^+^ T cells, regulatory T cells (Tregs) and myeloid cells, and display immunosuppressive TME as the parental melanoma tissues. Tumour infiltrating lymphocytes (TILs) expanded by interleukin‐2 (IL‐2) plus anti‐PD‐1 could significantly infiltrate into the immune organoids and facilitate tumour‐killing effects.^20^ Scognamiglio et al.^21^ generated immune organoids from a PD‐L1 positive chordoma sample, revealing the presence of cancer cells and PD‐1 positive lymphocytes. Evaluation of efficacy demonstrated that the PD‐1 inhibitor nivolumab significantly reduced organoid diameters and increased cell death.^21^ Similarly, the B7‐H3–antibody–DM1 conjugate displayed potent tumour‐killing effects in craniopharyngioma organoids equipped with patient‐matched immune systems.^22^ Organoid culture platforms have emerged as efficient and robust models that enable pretreatment assessments of immunotherapies and the identification of responsive patients.^23^ Using immune organoids helps identify non‐responsive patients, a crucial step in avoiding ineffective treatments and reducing the costs and toxicity associated with immunotherapy.^24^

Therefore, this review mainly summarised recent advances in using immune organoids for evaluating anti‐tumour immune responses. We explored the innovative methods to enhance PDTO‐based detection techniques, including bioluminescence methods, acoustic droplet systems, gene editing and others, while considering their applicability in clinical settings. In addition, the prospects and challenges of immune organoids were also discussed in this field.

## 2 DEFINITION OF PDTOS IN CANCER IMMUNE RESPONSES

The groundbreaking 2009 study by Sato et al.^25^ marked the initial successful establishment of intestinal organoids derived from leucine‐rich repeat containing G protein‐coupled receptor 5 (Lgr5)‐positive mouse intestinal stem cells. This research robustly expanded the applications of organoids, allowing them to be used in different tissues and species. These 3D multicellular organoids have demonstrated their ability to preserve the intricate architecture and functionality of their parent tissues.^26^, ^27^, ^28^ Organoids are grown under specific conditions, derived from stem cells or tissue‐specific progenitors, and autonomously develop to recapitulate the original histopathological traits and genomic profiles. The culture conditions should be optimised to promote efficient growth and maintain well‐defined physiological properties of organoids. Various factors such as genetic instability affect the differentiation of cells within the organoids. Maintenance of the genomic integrity of organoids over time during culture is crucial for accurately modelling the tumour biology and therapeutic response. Meanwhile, supplying appropriate growth factors, pathway inhibitors and culture substrates could expectedly control the growth and differentiation state.^29^, ^30^ Tüysüz et al.^31^ constructed the phospholipid‐ and cholesterol‐based carriers to enhance the stability and activity of purified Wnt3A. Subsequent administration of stabilised Wnt3A stimulated self‐renewal of stem cells and long‐term expansion of human organoids. Sato et al.^25^ pointed that the generation of intestinal organoids require the conditioned media that contain recombinant growth factors (epidermal growth factor, Noggin, Wnt3A and R‐spondin) as well as laminin‐rich Matrigel to support intestinal epithelial differentiation. Addition of small molecule inhibitors (ALK inhibitors and p38 inhibitors) and growth factors allowed the long‐term expansion of epithelial organoids carrying differentiated epithelial cell types and maintain their genetic stability.^32^

**FIGURE 1:**
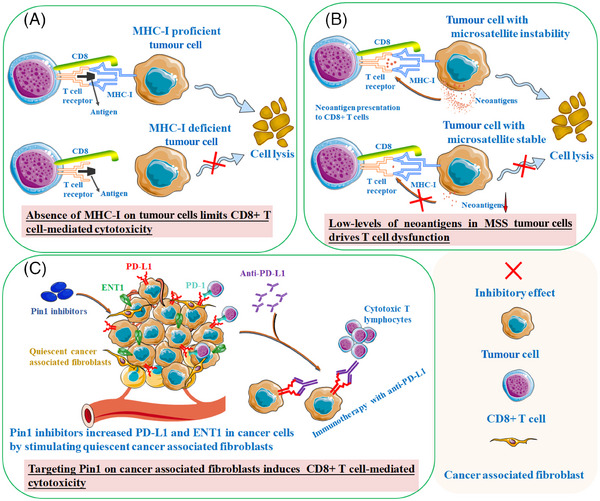
The significant challenge in poor immunotherapy is the limited cytotoxic capacity of T cells. (A) Absence of major histocompatibility complex class I (MHC‐I) on tumour cells limits the cytotoxic capacity of CD8+ T cells against tumour cells. (B) Low‐levels of neoantigens on tumour cells drives early T cell dysfunction and poor immunotherapy responses. 480 (C) Targeting Pin1 on cancer‐associated fibroblasts could drive CD8+ T cell‐mediated cytotoxicity.

Additionally, PDTOs can be efficiently expanded and cultured, reducing the need for large patient materials. They can also be preserved at low temperatures for long‐term storage, facilitating the creation of organoid biobanks to model development processes, disease progression and high‐throughput drug screening.^33^, ^34^, ^35^ The distinct genetic and phenotypic variances between normal tissue and tumour‐derived organoids allow the screening of therapeutic agents capable of selectively targeting tumour cells with minimal or no impact on non‐cancerous cells.^36^ Furthermore, the advancement of organoid culture systems has the potential to serve as a powerful tool to accurately predict a patient's response to therapy and tailor personalised treatment strategies.^37^, ^38^ In recent decades, organoid models derived from various common cancer types, such as glioma, ovarian and colorectal cancer, have been successfully cultivated.^39^, ^40^ Recent studies have expanded our scientific understanding of the functional advantages of PDTOs in immune responses, providing information for managing human cancers. A significant challenge in cancer therapy is the limited cytotoxic capacity of T cells against tumour cells due to the absence of the major histocompatibility complex class I (MHC‐I) molecules^41^ (Figure 1A). In particular, inhibition of ring finger protein 31 (RNF31) has been shown to sensitise MHC‐deficient organoids by disrupting tumour necrosis factor (TNF) signalling pathways and enhancing the cytotoxicity mediated by natural killer (NK) cells and CD8^+^ T cells.^42^ In the case of ex vivo neuroblastoma organoids, histone deacetylase inhibitors have shown the ability to alleviate the immunosuppressive microenvironment by down‐regulating PD‐L1 expression and up‐regulating MHC‐I expression in tumour cells. These effects effectively promote the recruitment of TILs and generate anti‐tumourigenic immune responses.^43^

Similarly, mitogen‐activated protein kinase inhibitors (MEKi) have been found to induce MHC‐I expression in lung cancer organoids, thus facilitating T cell‐mediated anti‐tumour immunity.^44^ Furthermore, combining immune checkpoint blockade (ICB) with MEKi has yielded higher levels of cytotoxicity by deactivating several oncogenic pathways in pancreatic cancer organoids.^45^ These preclinical organ models have proven invaluable in elucidating the mechanisms of immunotherapy and exploring potential combination treatment strategies.

## 3 CULTURE SYSTEMS TO MIMIC THE IMMUNE MICROENVIRONMENT

**FIGURE 2:**
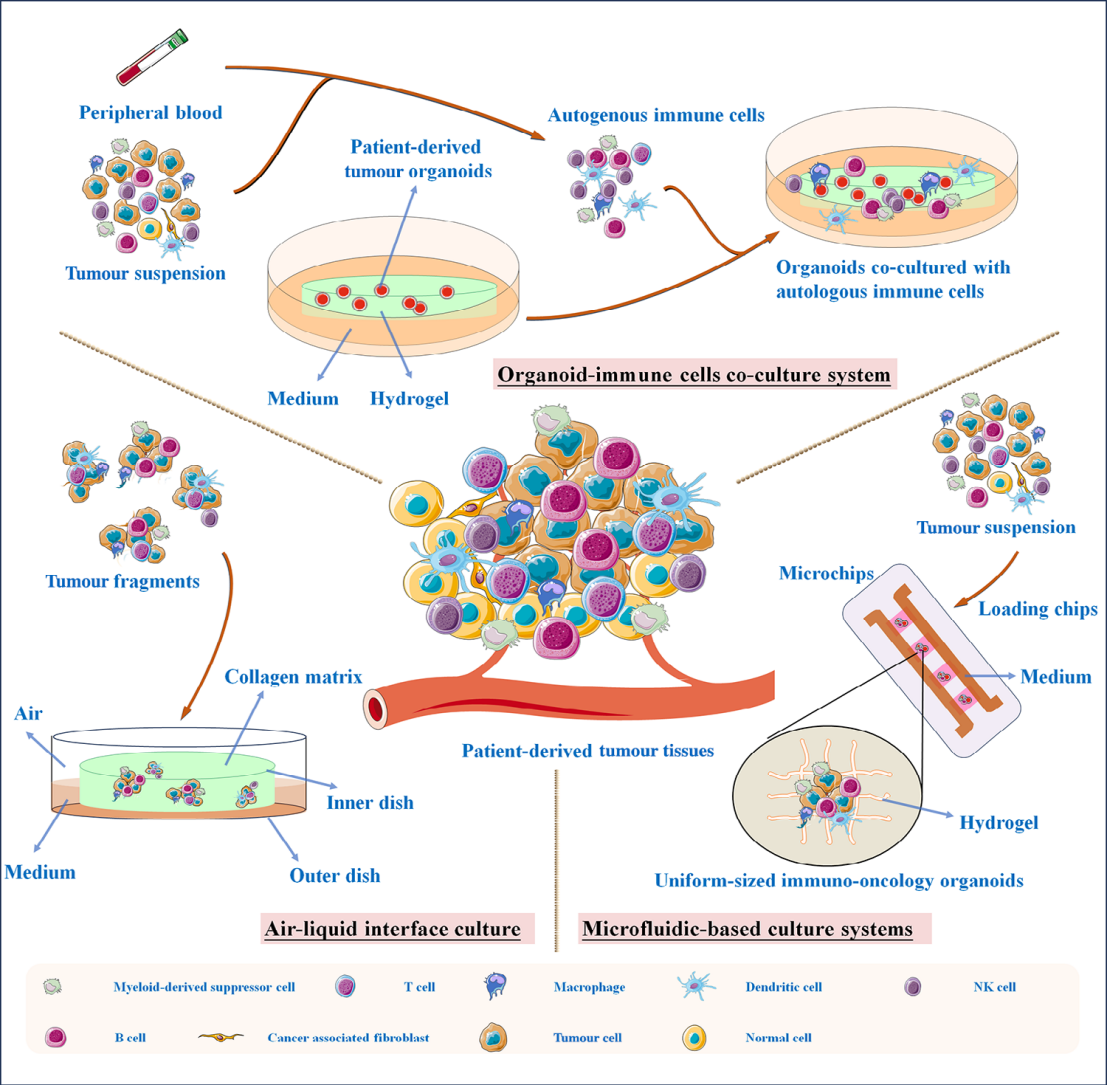
Schematic representation of culture systems for integrating tumour organoids with immune components. Three types of approaches have been utilised to generate the tumour immune microenvironment in patient‐derived tumour organoids (PDTOs), such as organoid‐immune cell co‐culture system, air–liquid interface and microfluidic‐based models.

Effective communication between peripheral and intratumoural components is vital for regulating tumour immunity. Unfortunately, traditional cancer organoids, due to their limited infiltration by immune populations, do not robustly reproduce the intricacies of immunological processes. This constraint hampers its potential for immuno‐oncology research.^46^ Undoubtedly, the promising solutions lie in integrating PDTOs with peripheral or lymphatic system immune components (Figure 2 and Table 3). These promising approaches would function as the crucial and suitable models for investigating patient‐specific tumour‐immune interactions.^47^, ^48^

**TABLE 3:** The culture systems of patient‐derived tumour organoids (PDTOs) to mimic the immune microenvironment.

Methods	Co‐culture system	Air–liquid interface	Microfluidic
Tissues processing	Tissues are physically and enzymatically dissociated	Tumour fragments	Small tumour suspension
Culture instrument	Any size of plate	Any size of plate	Microfluidic devices
Culture approach	Co‐culture of neoplastic cells with isolated immune components	Tumour fragments are seeded into collagen matrix, one surface submerged in liquid medium and the other exposed to air	Small tumour fragment‐collagen mixture is seeded into the 3D gel in central region of device; media are added into the channels located on both sides
Cell types	Tumour cells, autologous lymphocytes from peripheral blood or lymphatic system	Tumour cells, endogenous immune cells, non‐immune cells (native epithelial and stromal cells)	Tumour cells, stromal cells, autologous lymphocytes
Culture duration	Short‐term	Over a period of 1–2 months	Short‐term
Advantages	Easy to enrich and expand tumour organoids; to recruit tumour‐specific immune cells and evaluate the immuno‐oncology therapies	Preserving intricate endogenous immune and non‐immune cell populations, maintaining the original tumours’ genetic profiles, supporting functional differentiation of epithelial cells that are not normally exposed to ambient air	improving the consistency and precision of organoid models, retaining the cellular compositions and dynamic cell–cell interactions, real‐time monitoring and optimisation of organoid models
Limitations	Restricted to mimic the diversity and heterogeneity of immune populations. Exogenously added multiple immune cells	Restricted to long‐term mimic the circulating immune cells into tumour	Requiring specific microfluidic systems, cells quantities within microfluidic devices are too small

### 3.1 Organoid‐immune cell co‐culture system

One of the prevailing and easy methods for developing immune organoids involves the separate acquisition of autologous lymphocytes and organoids, followed by their co‐culturing.^49^ Incorporation of immune cells into tumour organoids could model the TME and deepen our understanding of the underlying mechanisms and biological behaviours within this environment. Recent studies have highlighted the effectiveness of co‐culture of neoplastic epithelium with immune components as a compelling strategy to address the deficiency of TILs in head and neck squamous cell carcinoma organoids.^50^ Co‐culture of cancer organoids with autologous lymphocytes from peripheral blood or lymphatic system represents a robust and unbiased approach to recruit tumour‐specific immune cells and evaluate the short‐term immuno‐oncology therapies.^51^ Moreover, in the co‐culture system, supplemented of various growth factors (R‐spondin and Wnt3A) and/or pathway inhibitors (TGF‐β inhibitor and p38 inhibitor) into the media could allow the cells to undergo self‐renewal and differentiation.^52^, ^53^

To create immune‐enhanced patient tumour organoids, surgically obtained melanoma tissues and lymph node biospecimens of the same patient were simultaneously integrated into an extracellular matrix (ECM) hydrogel.^54^ Meng's group, for instance, successfully generated CD8‐ or CD4‐positive organoid‐primed T cells through the co‐culture of PDTOs with autologous T cells from the peripheral blood of pancreatic cancer patients. This approach demonstrated its utility in evaluating the function of tumour‐specific TILs.^55^ Similarly, Dijkstra et al.^56^ established and validated a co‐culture platform to isolate tumour‐reactive lymphocytes in a personalised manner. Their work revealed that tumour organoids cultured with peripheral blood lymphocytes could effectively generate tumour‐reactive CD8^+^ T cells, thus facilitating adaptive anti‐tumour immunotherapy.^56^

Using CRISPR‐mediated mutagenesis, Coelho et al.^57^ systematically explored the genetic landscapes of interferon γ (IFN‐γ) in colorectal cancer tumour organoids co‐cultured with matched tumour‐reactive T cells. Their findings revealed that missense mutations in Janus kinase 1 (JAK1) could enhance or decrease the activity of IFN‐γ signalling pathway, thus influencing the cytotoxicity of autologous tumour‐reactive T cells within organoids. In models involving co‐culture of gastric cancer organoids with immune cells, inhibition of epidermal growth factor receptor 2 (HER2) suppressed PD‐L1 expression and activated CTLs by disrupting the AKT–mTOR signalling pathway, ultimately leading to cell death within organoids.^58^ Furthermore, a dynamic 3D co‐culture system was developed to replicate the reciprocal interactions between dendritic cells (DCs) and cancer cells in organoids derived from metastatic colorectal cancer.^59^ Upon anti‐PD‐1 treatment, direct cell–cell contact effectively facilitated TIL‐mediated anti‐tumour immune responses within organoids.^60^ Consequently, this organotypic co‐culture platform offered innovative and direct insights into the phenotypes and behaviours of immune cells driven by tumour organoids. Moreover, the co‐culture of PDTOs with autologous lymphocytes provides a unique evaluation technique for investigating the interplay between cancer cells and immune cells in individual patients.

However, the addition of single immune cell types in co‐culture system may not completely mimic the intricate interactions between various immune populations. To solve this problem, Chakrabarti's group^61^ constructed a complex model to co‐culture activated CTLs and DCs within gastric cancer organoids. This reconstitution approach expectedly recapitulated the interactions of tumour‐immune and immune‐immune cells and significantly enhanced the anti‐tumour immunity.

### 3.2 Air–liquid interface

Although organotypic co‐culture systems incorporate immune cells, they often fall short in capturing the diversity and heterogeneity of tumour‐infiltrating immune cells within the microenvironment. Consequently, there is a pressing need to develop innovative strategies for modelling immune organoids. A novel organoid generation platform based on the air–liquid interface (ALI) has been successfully established and used as an assessment tool for cancer immune responses.^62^, ^63^ In ALI system, tumour fragments are introduced into a collagen matrix where basal surface of the fragments are submerged in a culture medium and the apical surface is exposed to air.^64^ Remarkably, ALI approaches are able to support the functional differentiation of 3D multilayered cells that do not necessarily have apical access to air, such as epithelia originating from intestinal, and excellently maintain their functionality.^65^ More importantly, PDTOs in ALI systems can accurately mimic the histology and genetic profiles of original tumours over a period of 1−2 months. Different from co‐culture system, ALI PDTOs also preserve the intricate tumour‐infiltrating immune cells, such as T cells, B cells and DCs, along with non‐immune constituents (native epithelial and stromal cells).^66^ Even though the vasculature in ALI effectively mimics the circulation of immune cells,^67^ their exhaustion is still a challenge.

To support these points, patient‐derived renal cell carcinoma organoids, established through ALI technology, have demonstrated precise preservation of T cells, cancer‐associated fibroblasts (CAFs) and CD31^+^ endothelial cells.^68^ Using the collagen‐based ALI method, Esser's research group successfully generated PDTOs from resected tumour tissues from patients with renal cell carcinoma. These ALI organoids effectively preserved multiple immune and stromal cells.^69^ The next crucial step involves investigating the role of ALI‐generated organoids in determining the efficacy of immunotherapy. PDTOs, which retain the original tumour T cell receptor spectrum, can functionally model PD‐1/PD‐L1‐dependent therapy. Inhibitors targeting the PD‐1/PD‐L1 signalling pathway have been shown to activate tumour‐reactive TILs and induce anti‐tumour cytotoxicity.^70^ When treated with the PD‐1 inhibitor toripalimab, ALI‐generated organoids exhibited a significant increase in the CD8^+^/CD4^+^ T cell ratio and a higher number of apoptotic cancer cells.^68^ These findings suggest that ALI organoids, which incorporate immune and other stromal components, can be an effective preclinical strategy to monitor responses to immunotherapy and guide personalised medicine.

### 3.3 Microfluidic‐based models

Various microfluidic‐based devices, such as microfluidic organs‐on‐chips,^71^ have been developed to recreate the immune microenvironment and assess immunotherapeutic responses in human cancers. These microfluidic systems incorporate multiple sensors and actuators into a single microchip, enabling real‐time monitoring and optimisation of detection parameters and culture conditions during organoid cultivation.^72^, ^73^ These technological advances improve the consistency and precision of organoid models, highlighting their potential applications in evaluating immune‐tumour interactions.^74^ Ao et al.^75^ established a microfluidic‐based mini‐tumour chip model to examine the dynamic interactions between cancer cells and the immune system. They successfully generated 960 homogeneous mini‐tumour spheroids on these chips by uniformly infusing dissociated tumour cells into isolated microfluidic well‐arrays. These uniform‐sized 3D mini‐spheroids, formed by the self‐assembly of cells in suspension or on non‐adherent surfaces, could mimic the cellular compositions and dynamic cell–cell communication observed in host tumours. Especially, organotypic tumour spheroids derived from patients contain the cancer cells and autologous immune/stromal cells^76^, ^77^ and represent a candidate system mimicking the actual TME.^78^ However, they lack the complex tissue architecture found in organoids. Additionally, the organ‐on‐a‐chip model, also known as co‐culturing organoids in microfluidic systems, demonstrated exceptional predictive values for assessing the therapeutic efficacy of anti‐PD‐1 treatment as early as four hours after drug injection, showcasing its clinical potential to identify responsive patients during immunotherapy rapidly.^79^, ^80^

Recently, a droplet emulsion microfluidics system has been developed to efficiently generate hundreds of micro‐organospheres, the small spherical structures formed by the aggregation of cells in suspension culture, from various patient tissues.^81^ This system successfully captures and manipulates multiple types of cells from the original tissues, including stromal cells and lymphocytes, but typically smaller and less complex than traditional organoids. Notably, the droplet emulsion microfluidics system offers several appealing advantages, such as precise temperature control and minimal dead volume, facilitating the rapid and efficient expansion of PDTOs for high‐throughput immunotherapeutic drug screening. Furthermore, Wu et al.^82^ rapidly generated thousands of clusters of organotypic cells from breast tumour tissues using another highly biocompatible, contactless, label‐free fluid technology. These uniformly sized organotypic cell clusters, formed by the aggregation of cells in conditioned culture, could exhibit some specific tissue‐like structures and functions, but typically less organised and complex than traditional organoids. Most notably, the integration of time‐lapse cell imaging and these organotypic cell clusters accurately and consistently recreated the dynamics of cancer‐immune interactions and their responses to immuno‐oncology therapies.

Microfluidic technologies have allowed the co‐culture of PDTOs with autologous immune cells within continuously perfused chambers, providing structural and functional support to recreate physiological conditions of the human body.^83^ For example, an in vitro microfluidic device was used to co‐culture breast cancer cells with low PD‐L1 expression alongside mesenchymal stem cells (MSCs). This study demonstrated that MSCs release inflammatory cytokines, particularly the C‐C motif chemokine ligand 5 (CCL5), which significantly stimulate the expression of PD‐L1 in breast cancer cells, thus creating an inhibitory immune microenvironment.^84^ In contrast, administering cytokine inhibitor pirfenidone reduced PD‐L1 expression levels and alleviated immune‐suppressive capacity.^85^

Another microfluidic platform designed to mimic the interstitium of the tumour consists of 3‐µm channels loaded with conditioned medium, GP33‐targeted CD8‐positive T cells and GP33‐positive cancer cells, respectively. Under the influence of hydrostatic pressure, GP33‐antigen‐specific T cells were observed to rapidly transmigrate to GP33^+^ hepatic cancer cells, inducing cell apoptosis.^86^ In a separate study, Park et al.^87^ introduced the CACI‐IMPACT platform, which used an injection‐moulded plastic array culture system to facilitate the co‐culture of cytotoxic lymphocytes with cancer cells and explore the anti‐cancer capacities of cytotoxic lymphocytes in a 3D microenvironment. The dense hydrogel framework promoted NK‐92 cell infiltration into cancer cells, enhancing the NK‐92‐mediated tumour‐killing effects within this platform. Similarly, by co‐culture of NK‐92 cells with pancreatic cancer cells (MIA PaCa‐2) or breast cancer cells (MCF‐7/MDA‐MB‐231), a 3D tumour spheroid microarray was successfully developed for high‐throughput screening of NK‐mediated anti‐cancer immunity.^88^, ^89^

These findings above‐mentioned collectively demonstrate the facility and high‐resolution of microfluidic technologies in discovering personalised immunotherapy options for cancer patients. However, the quantities of cells within microfluidic devices are too small for additional immune‐associated assays.^90^ Considering that different immune cells thrive in different culture conditions,^91^ multiple channels for separate immune population should be well integrated within one microfluidic device. Currently, these fabricated models are used primarily in engineering and biology laboratories. There is a growing effort to adapt these models to bridge the gap between preclinical research and clinical practice.

## 4 USING ORGANOIDS TO STUDY THE IMMUNO‐GENOMIC CHARACTERISTICS

Immunotherapies have emerged as validated and critical essential strategies for treating cancer patients. However, considering that immunotherapy has shown success and efficacy in only a select group of patients, it is urgent to accelerate our understanding of the complex immune characteristics within the TME.^92^ To address these challenges, patient‐specific model systems, such as immune organoids, hold promise as a safe and promising alternative for measuring immunological signatures.

Immune cell profiling provides proof‐of‐concept information to assess agents’ responses. Existing organoid models allow longitudinal characterisation of immune cell changes within tumour cells or the TME after drug treatment.^93^, ^94^ Pairwise analyses of intrinsic immuno‐genomic characteristics have revealed similar immunogenetic signatures and histopathological features between organoids derived from colorectal cancer‐derived and their corresponding primary tumours.^95^ For example, He's group^96^ established patient‐derived lung metastatic osteosarcoma organoids (OSOs) that maintained histological characteristics and expressed osteosarcoma biomarkers (Vimentin and Sox9). They also identified similar distributions of T lymphocytes in the microenvironment of OSOs and osteosarcoma tissues of patients. Co‐culture systems involving organoids of pancreatic ductal adenocarcinoma and peripheral NK cells exhibited concordant phenotypes and functions of NK cells within surgically removed tumours. Upon loss of inhibitory signalling, NK cells in organoids and primary tumours showed low‐CD16 low‐CD57 phenotypes and improved cytotoxicity against human cancers.^97^ Graney and colleagues^98^ confirmed the presence of early memory B cell‐like phenotype in immune organoids after pre‐treatment with IL‐9 and IL‐21, highlighting the potential of organoid models for the future investigation of B cell‐mediated anti‐tumour immunity.

Recent advances in efficient organoid cultivation have established a solid foundation for tracking immunophenotypic features after immunotherapies.^99^, ^100^, ^101^ Moreover, single‐cell sequencing technologies have rapidly facilitated the deeper analyses of immune characteristics in organoids. For example, single‐cell RNA sequencing, combined with fluorescence‐activated cell sorting, has indicated that immune organoid models expectedly preserve the TIL clonotypes and immune diversity from the corresponding biopsies.^102^ Similar methods could also be employed to comprehend, predict and optimise the ICB response in immune organoids. In bladder cancer organoids depleted of Trp53, phosphatase and tensin homolog, and retinoblastoma 1, single‐cell sequencing unveiled distinct landscapes of tumour‐infiltrating immune cells between responders and nonresponders to ICI. In responders treated with anti‐PD‐1 antibodies, there was a marked increase in immune cell infiltration, including tumour‐associated macrophages and T cells.^103^ Similarly, in melanoma organoids co‐cultured with peripheral blood mononuclear cells (PBMCs), ICI nonresponders exhibited an early increase in FKBP51s^+^PD‐L1^+^ monocytes, promoting pro‐tumourigenic M2 polarisation and subsequent resistance to PD‐1 blockade.^104^ Thus, with the assistance of single cell technology, immune organoids could accelerate the discovery of cell‐intrinsic and cell‐extrinsic targets for cancer immunotherapy within microenvironmental components.

A novel multispectral and image‐based 3D platform called BEHAV3D has recently been developed, enabling real‐time and simultaneous measurement of the action modes of cellular immunotherapy in large amounts of PDTOs. Using BEHAV3D, Dekkers et al.^105^ identified a surprising ‘super engager’ cluster comprising cytotoxic CD8^+^ TILs in breast cancer organoids. Furthermore, the administration of type I interferon (IFN‐I) improved super‐engagers' ability to eliminate nonresponsive organoids.^105^ Together, these findings underscore the utility of organoid culture systems in defining the immune microenvironment's composition and architecture and guiding personalised anti‐cancer therapy.

## 5 T CELL‐MEDIATED IMMUNITY AGAINST CANCER

To effectively monitor T cell‐mediated immunity, it is crucial to develop technological platforms to assess the quality and magnitude of T cell responses and understand the underlying determinants. 3D platforms such as PDTOs allow us to analyse individual T cell‐mediated immunotherapy responses, significantly deepening our critical understanding of factors that influence therapeutic efficacy.^106^, ^107^ It should be noted that the immunoregulatory components within the microenvironment of these 3D models profoundly affect the infiltration of immune cells, particularly T cells and modulate their cytotoxic activities against tumour cells.^108^, ^109^

Tumour‐infiltrating CD8^+^ T cells are the primary immune cells responsible for immune protection against cancers and constitute the foundation of current successful immunotherapy.^110^, ^111^ The amino acid oxidase IL‐4‐induced‐1 (IL‐4I1) has recently been identified as a pro‐tumourigenic factor. When co‐cultured with organoids that overexpress IL‐4I1, tumour‐reactive CD8^+^ T cells showed up‐regulated exhaustion‐associated surface biomarkers (PD‐1 and T cell immunoglobulin and mucin domain‐containing‐3 (TIM3)) and impaired cytolytic activity against cancer cells.^112^ In gastric cancer organoids co‐cultured with PBMCs, inhibition of nicotinamide phosphoribosyltransferase using genetic and pharmacological approaches favoured the infiltration of CD8^+^ T cells into the tumour by disrupting the balance of adenosine triphosphate and generally inducing apoptotic cell death.^113^ Simultaneously, treatment with atractylenolide I substantially improved MHC‐I‐mediated antigen presentation and improved CD8^+^ T cell functions, ultimately causing immunogenic cell death in organoids of colorectal cancer.^114^

The effectiveness of immunotherapy is based on the ability of T cells to target neoantigens. Cancer patients with a high burden of neoantigens tend to benefit from immunotherapy drugs.^115^ Cancer cells can produce newly formed mutant peptides called neoantigens under certain stimulating conditions, such as dysregulated post‐translational modifications, disordered RNA splicing and viral infections. Multiple omics technologies have been used, including whole genome sequencing‐based human leukocyte antigen (HLA) class‐I score systems^116^ and mass spectrometry immunopeptidomics,^117^ to assess the immunogenic potential of neoantigens in tumour tissues and their corresponding organoids. The results prove organoids preserve the comparable immunogenicity of neoantigen peptides found in parental tumours. These neoantigens, characterised as non‐synonymous tumour‐specific mutations, can be recognised by stimulated endogenous T cells, leading to an anti‐cancer immune response.^118^, ^119^ In this regard, organoid‐killing assays have shown that high‐affinity neoantigens can activate tumour‐specific CD39^+^CD8^+^ T cells, thus improving therapeutic outcomes in anti‐PD‐1 therapy.^120^

In an orthotopic organoid model of pancreatic cancer, combination therapy involving CD40 antibodies (CD40Ab) and irreversible electroporation (IRE) improved the recognition of neoantigens by DCs, subsequently triggering a robust CD8^+^ T cell response that facilitated tumour control.^121^ Simultaneously, nanoparticles encapsulating innate immune stimulants proved sufficient to increase the activation of neoantigen‐specific CD8^+^ T cells, resulting in robust suppression of tumour progression in PDTOs.^122^ Furthermore, patients with microsatellite‐stable colorectal cancer expressed low levels of neoantigens, leading to early T cell dysfunction and poor ICB responses^123^ (Figure 1B). In treatment‐resistant colorectal cancer organoids, Gonzalez‐Exposito et al.^124^ made a surprising discovery that inhibiting the WNT/β‐catenin pathway had the potential to trigger T cell‐mediated killing and sensitise cancer cells to CEA‐targeting antibodies by upregulating CEA expression.

These studies have demonstrated the potential of organoid generation as a technology for reprogramming T lymphocytes, thereby enhancing tumour cell sensitivity to T cell‐mediated cytotoxicity.^125^ These findings support that 3D organotypic models can influence T cell metabolism at personalised levels.

## 6 ADVANCES IN ADOPTIVE CELL‐BASED IMMUNOTHERAPY

In recent decades, ACTs utilising genetically engineered lymphocytes, such as chimeric antigen receptor (CAR)‐modified T cells and NK cells, have achieved remarkable success in cancer immunotherapy.^126^, ^127^ By reengineering the immune system to recognise tumour‐associated antigens (TAAs), ACTs hold great promise for anti‐tumour reactivity and significantly improve patient survival.^128^ Although ACTs have made breakthroughs in hematologic malignancies, their effectiveness in treating solid tumours remains limited.^129^ Furthermore, several challenges must be addressed to transform this treatment modality into an innovative anti‐cancer strategy in clinical practice, including off‐tumour toxicity and frequent relapse after ACTs.^130^, ^131^

Increasing evidence supports the development of CAR cells targeting tumour antigens for immunotherapy. In this context, PDTOs demonstrate attractive potential as personalised evaluation models for ACTs.^132^, ^133^ At the same time, several technologies, such as BEHAV3D^134^ and luciferase‐based imaging,^135^ have proved the powerful strategies to identify the characteristics and treatment efficacy of CAR cells in immuno‐organoids. A recent review^136^ has also confirmed that CAR T cells targeting TAAs effectively killed TAA^+^ cancer cells and organoids, consequently extending the patients’ survival. In MET‐CAR T co‐cultured organoids derived from gastric carcinoma patients, MET‐CAR T cells exhibited improved immunotherapy efficacy against MET‐amplified tumour cells and organoids.^137^ CAR T cells effectively targeted MUC1, a TAA closely related to tumour progression and therapeutic response, leading to durable and specific immune cytotoxicity in MUC1^+^ organoids but not MUC1^−^ organoids.^138^ Birinapant, an inhibitor of apoptosis protein antagonist, sensitised CAR T co‐cultured glioma organoids to cytokines released from EGFRvIII‐targeted CAR T cells, including TNF.^139^ Importantly, B7‐H3‐targeted CAR T cells overexpressing the CCL2 receptor, CCR2B, exhibited an excellent ability to penetrate the blood‐brain barrier, enhancing the effectiveness of ACTs against brain metastases in human cancers.^140^ Recently, a fraction of CD39^hi^ subpopulations of CD8^+^ T cells with exhausted features has been identified to block the cytotoxic CTL response in gastric cancer organoids.^141^ Overexpression of SMAD7, an inhibitor of TGF‐β signalling, in HER2‐targeted CAR T cells significantly suppress the TGF‐β‐triggered exhaustion and displayed sustained T cell‐mediated tumouricidal capacity in PDTOs.^142^ Therefore, although T cell exhaustion poses a challenge for long‐term culture and application of organoids, supplementation with some factors that block exhaustion could potentially improve the cytolytic efficacy of CAR T cell therapy against cancers.

Recently, CAR NK cell therapies also display the excellent tumour‐killing effects. It is known that the ligands of C‐X‐C motif receptor 2 (CXCR2) are highly secreted by human cancer cells, including pancreatic cancer. CXCR2‐augmented CAR NK cells could be strongly infiltrated into tumour sites, consequently reversing the immunosuppressive TME and enhancing the lysis of CXCR2 ligand‐secreted cancer organoids.^143^ CD19 CAR‐iNK cells could targeted recognised the CD19‐positive pericytes in glioblastoma microenvironment. The presence of pericytes notably facilitated the migration of CD19 CAR‐iNK cells towards glioblastoma cells and exhibited strong growth inhibition effects.^144^ Schnalzger et al.^135^ demonstrated the activity of EGFRvIII‐targeted CAR NK‐92 cells against EGFRvIII^+^ colorectal cancer organoids. In this study, they reported that addition of CAR NK‐92 cells was able to lyse the patient‐derived colon organoids in a co‐culture system with Matrigel‐coated layer. Moreover, CAR‐NK‐92 cells combined with ICIs could further increase the host T‐cell defence against cancers.^145^

In ACTs, several alternative approaches offer attractive advantages that can enhance cytotoxic efficiency and address current limitations. One such technique involves using CAR‐engineered PSC lines to produce allogeneic CAR cells, potentially broadening patient access to ACTs.^146^ To test this hypothesis, Li et al.^147^ developed an artificial thymic organoid platform to effectively guide the differentiation of CAR^+^iPSCs into highly functional CAR T cells. In particular, iPSC‐derived CAR T cells exhibited functional profiles comparable to traditional CAR T cells, including antigen‐specific recognition, cytokine secretion and cytotoxicity.^148^ Moreover, promoting the formation of CAR clusters offers the potential to optimise CAR cell therapy against cancers.^149^ In this context, the trivalent tetrahedral DNA nanostructure on the surface of CAR T cells rapidly enhances the aggregation of tumour‐infiltrating T cells, improving their killing activity. This ultimately leads to apoptosis‐inducing effects and reduced side effects in liver cancer organoids.^150^ Despite the promise and significance of these systems, there are various critical considerations. These include how to integrate them into clinical applications and optimise clinical management.

It is reasonable to hypothesise that these 3D organotypic platforms present novel opportunities for screening personalised cell‐engineered therapies. Delving into the underlying mechanisms of these distinctive strategies may have significant clinical application potential.

## 7 EVALUATE THE EFFICACY OF ICB

Although ICB has shown promising therapeutic efficacy in various cancer types, many patients do not respond to this treatment. Primary factors contributing to treatment failure include low abundance of immunogenic antigens, compromised immune cell function and the secretion of immunosuppressive factors.^151^, ^152^ The need to determine whether patients would benefit from checkpoint inhibitors has led us to seek more effective experimental prediction methods. Predicting tumour responses to immunotherapy agents can also guide treatment decisions and extend overall survival. A groundbreaking multiorgan‐metastatic organoid has been created that successfully demonstrates the metastasis‐inhibiting effect of CTLA‐4 inhibitors.^153^ This proof‐of‐concept study highlights those immune organoids significantly advance predicting response to ICB therapy.

**FIGURE 3:**
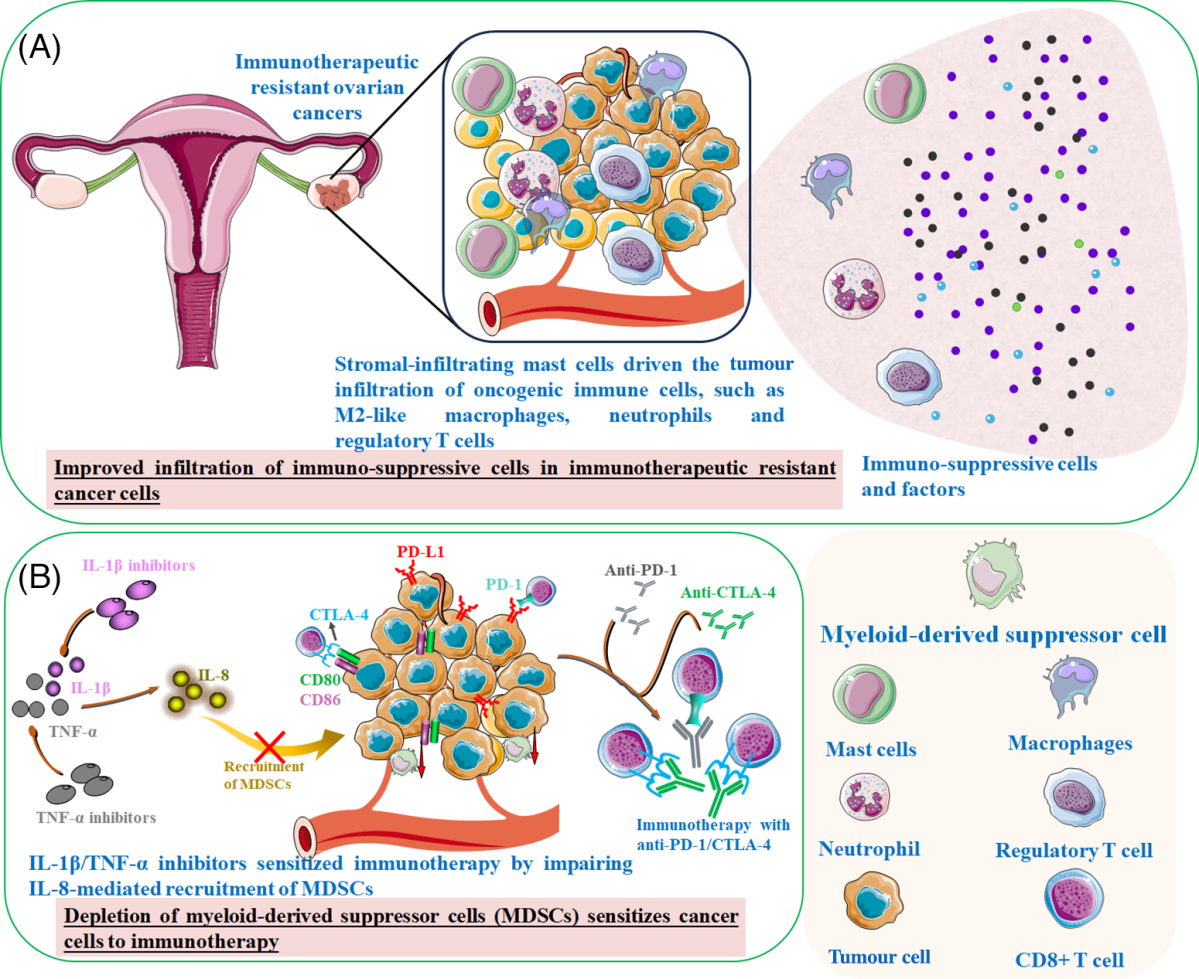
An abundance of immuno‐suppressive cells has been associated with immunotherapeutic resistance. (A) Improved infiltration of immuno‐suppressive cells (myeloid‐derived suppressor cells, cancer‐associated fibroblasts, M2 macrophages, etc) in immunotherapeutic resistant cancer cells. (B) Depletion of immuno‐suppressive cells could sensitise cancer cells to immunotherapy.

Mesenchymal stem/stromal cells (MSCs) within the TME are well documented for their influence on ECM formation, contributing to tumourigenesis and therapy resistance.^126^, ^127^ This concept is supported by observations of high mast cell levels in organoids that do not respond to anti‐PD‐1 therapy.^154^ An abundance of stromal‐infiltrating mast cells has been associated with immunotherapeutic resistance in high‐grade serous ovarian cancer‐derived organoids. This resistance is characterised by increased infiltration of oncogenic immune cells, including M2‐like macrophages, neutrophils and Tregs and a decrease in anti‐tumour immunity (Figure 3A). CAFs, another population of stromal cells within the TME, play a crucial role in the immune response of cancer cells.^155^ The inhibitors that target peptidyl‐prolyl cis/trans isomerase NIMA‐interacting 1 (Pin1) have been shown to disrupt the immunotolerant microenvironment by significantly deactivating CAFs. The pancreatic ductal adenocarcinoma organoids co‐cultured with CAFs displayed that Pin1 inhibitors could reinforce the effectiveness of anti‐PD‐L1‐mediated eradication against human pancreatic ductal adenocarcinoma^156^ (Figure 1C). Furthermore, MSC infiltration can also facilitate the expansion of myeloid‐derived suppressor cells (MDSCs), ultimately creating an immunosuppressive microenvironment that hampers the response to ICB^157^ (Figure 3B). Chen et al.^158^ observed a negative correlation between tumour‐infiltrating MDSCs and the response to anti‐PD‐1 therapy in PDTOs. Co‐culture of PDTOs and autologous immune cells revealed that depleting MDSCs could enhance the sensitivity of gastric cancer organoids to anti‐PD‐L1‐induced cell death.^159^ Furthermore, blocking pro‐inflammatory cytokines IL‐1β and TNF‐α impaired IL‐8‐mediated chemotactic recruitment of MDSCs and improved responsiveness to anti‐PD‐1/anti‐CTLA‐4 checkpoint inhibitors.^160^ However, additional research is still required to thoroughly investigate MSC reprogramming within organoid platforms to achieve satisfactory outcomes with immunotherapy.

**FIGURE 4:**
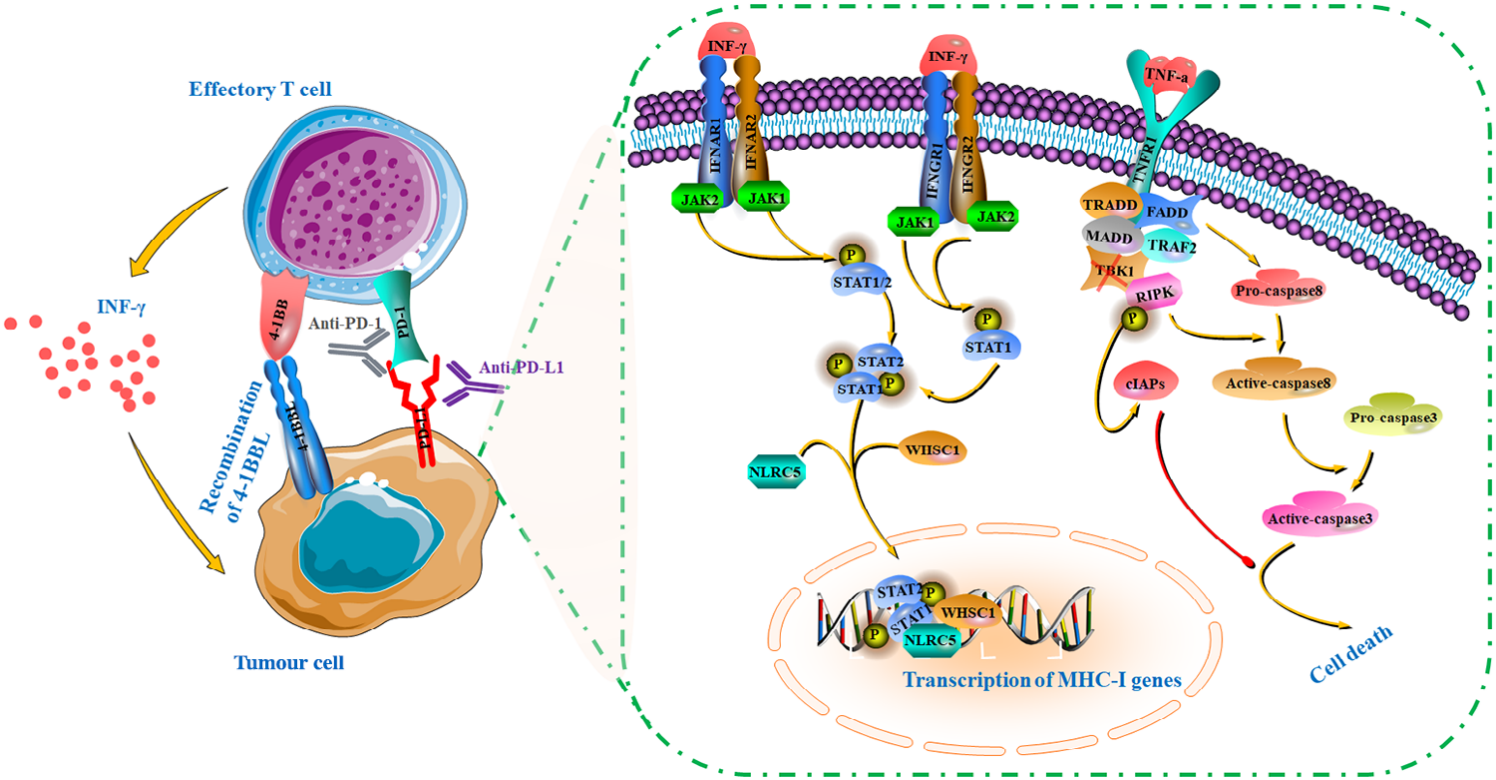
Alterations of interferon γ signalling pathway in immune organoids control the anti‐cancer immune response. The schematic illustrations indicating the landscapes of interferon γ (IFN‐γ) in organoids co‐cultured with immune cells. Generally, IFN‐γ displays the excellent potential in regulation of cellular immunity and subsequently, stimulation of anti‐tumour immune‐response. Generally, immune checkpoint blockade (ICB) therapy tends to elevate IFN‐γ levels, thus amplifying the anti‐tumour immune response through enhanced IFN‐γ‐induced MHC‐I antigen presentation. Activated IFN‐γ signalling could enhance MHC‐I gene expression by stimulating the activity of Wolf‐Hirschhorn syndrome candidate 1 (WHSC1). In addition, aberrant Janus kinase 1 (JAK1) could enhance or decrease the activity of IFN‐γ signalling pathway, thus influencing the cytotoxicity of autologous tumour‐reactive T cells within organoids.

A growing body of evidence has strongly suggested the excellent potential of IFN‐γ signalling in cancer immunotherapy (Figure 4), encompassing immune clearance and escape.^161^ Generally, ICB therapy tends to elevate IFN‐γ levels, thus amplifying the anti‐tumour immune response through enhanced IFN‐γ‐induced MHC‐I antigen presentation. Activated IFN‐γ signalling, in particular, can enhance MHC‐I gene expression by stimulating the activity of histone dimethyltransferase Wolf‐Hirschhorn syndrome candidate 1 (WHSC1). In models of colorectal cancer organoids, loss of WHSC1 resulted in reduced MHC‐I levels, thus limiting the therapeutic benefits of ICB driven by IFN‐γ signalling.^162^ Sun et al.^163^ further conducted organoid assays, demonstrating that the knockdown of TANK binding kinase 1 could overcome resistance to PD‐1 blockade treatment by inducing the release of inflammatory cytokines, including IFN‐γ. An's research group^164^ revealed that SMAD4‐deficient gastric cancer organoids resisted ICB monotherapy due to the reduced expression of 4‐1BBL, a costimulatory receptor in T cells. The reintroduction of 4‐1BBL facilitated IFN‐γ release in PDTOs, resulting in more effective tumour cell elimination.^165^ However, sustained activation of IFN‐γ signalling can lead to the synthesis of immune checkpoint inhibitory molecules, which activate other immune‐suppressive signalling pathways. In vitro and in vivo models have indicated that prolonged IFN signalling significantly confers resistance to ICB by upregulating multiple inhibitory receptors in TILs.^166^

In conclusion, these organotypic models have illuminated their potential as valuable systems for obtaining patient‐specific responses and facilitating personalised ICB‐based therapies. However, the utilisation of immune organoids to distinguish personalised responses to immunotherapy needs to be reproduced and confirmed in more validation cohorts.

## 8 MICROBE‐BASED CANCER IMMUNOTHERAPY

The use of microbial composition to investigate the interactions between the immune system and cancer cells has received growing interest. Microbial infections significantly influence cancer therapy by shaping the therapeutic effectiveness and toxicity of immuno‐oncology agents.^167^ Furthermore, the influence of the microbiota on the immune response to cancer underscores the potential benefits of constructing microbial networks for clinical cancer management.

Various microorganisms are found at elevated frequencies in cancer patients. In particular, recent research has confirmed the presence of CD8^+^ T cell epitopes, such as HLA‐A2 epitopes, within solid tumours, particularly human endogenous retroviruses.^168^ Moreover, patient‐derived glioma tissues and organotypic models have been shown to express coronavirus entry factors (angiotensin converting enzyme 2, transmembrane serine protease 2 and neuropilin 1).^169^ These discoveries suggest that host–microbe interactions are significant for oncogenesis, progression and treatment. For example, Allen et al.^170^ discovered that co‐culture of colon cancer organoids with Enterotoxigenic *Bacteroides fragilis* could trigger a host immune response and promote tumour formation. Shelkey et al.^171^ investigated various bacterial metabolites derived from the host–microbiome system and discussed their impact on anti‐tumour immunity. In their study, administering bacterial‐derived metabolites to breast cancer organoids significantly improved ICB‐induced apoptotic cell death by reducing immunosuppressive factors in MDSCs and increasing FAS‐L expression in CTLs. In PD‐L1 blockade, elevated *Fusobacterium nucleatum* levels increased IFN‐γ concentrations and attracted IFN‐γ^+^CD8^+^ TILs in colon cancer organoids.^172^ Similarly, targeting hepatitis B virus surface proteins by CAR T cells significantly promoted CD8^+^ T cell infiltration and IFN‐γ secretion, increasing anti‐tumour activity in hepatocellular carcinoma organoids.^173^

Various genetically engineered viruses have emerged with technological advances demonstrating their potential for immunomodulatory therapy. Engineered oncolytic adenoviruses, such as Ad‐Cab, have shown the ability to activate NK cells and neutrophils simultaneously, significantly increasing tumour cell death.^174^ Recently, Rangsitratkul et al.^175^ introduced a new oncolytic vesicular stomatitis virus, VSVd51–hGM–CSF, which contains genetic information for human granulocyte‐macrophage colony‐stimulating factors. Infection of organotypic models of bladder cancer with VSVd51–hGM–CSF significantly improved the infiltration of CD56^+^ NK cells and CD8^+^ T cells into the tumour, promoting immunogenic cell death.

In summary, exploring host–microbe interactions has emerged as a new research frontier that aims to identify more effective tumour immunotherapies. 3D platforms, particularly tumour organoids encapsulating immune components, allow for the close monitoring and elucidation of microbe‐mediated cytotoxic effects.

## 9 DISCUSSION

**FIGURE 5:**
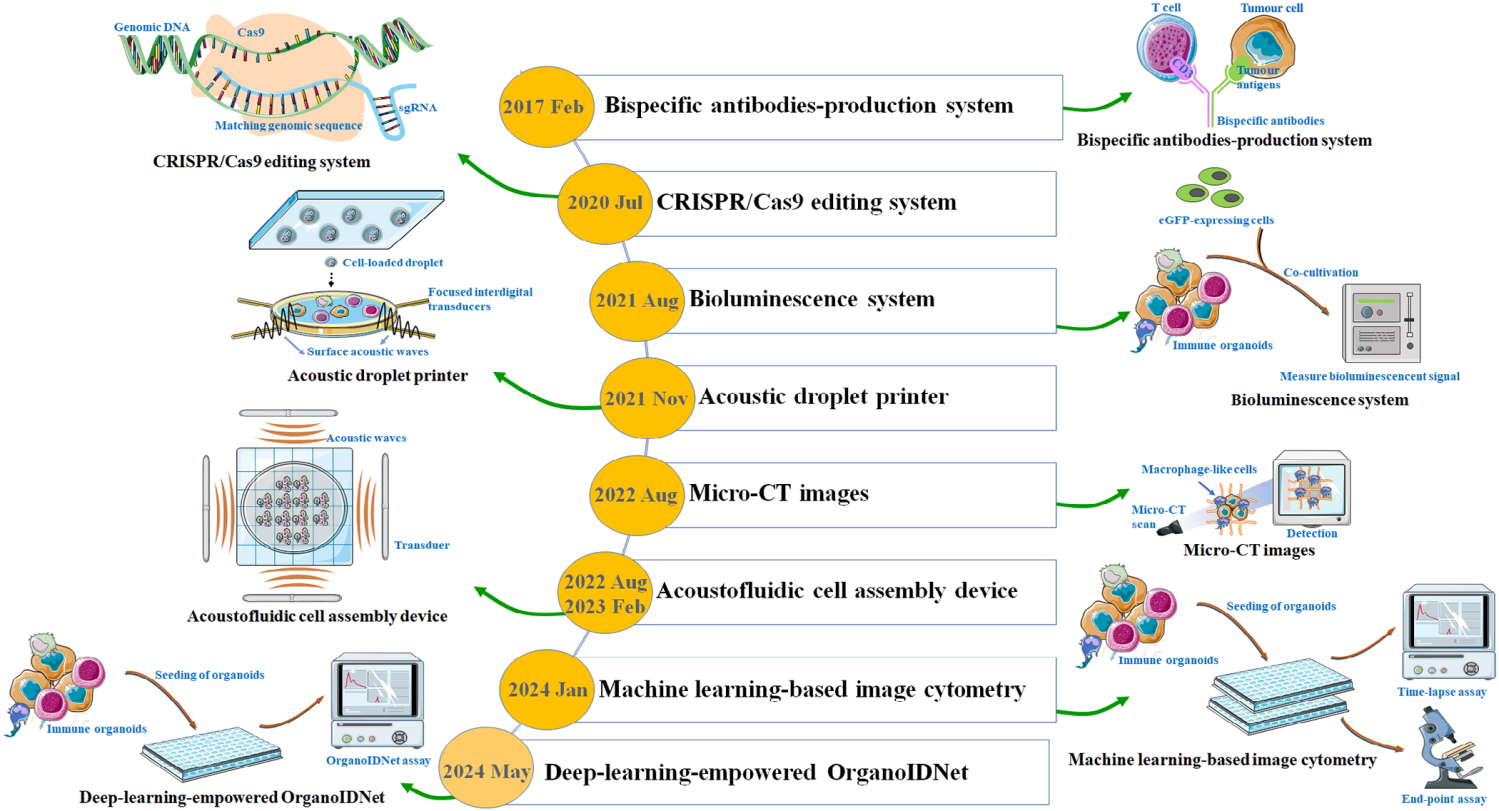
The application of novel biomaterials or synthetic technologies for immune organoids. These representative examples highlighted the potential of emerging technologies to enhance the functional relevance of immune‐tumour organoids, offering valuable alternative methods to investigate communication between tumours and immune cells. (I) The bispecific‐antibody secreted immunotherapeutic organoids is feasible to sustainedly generate therapeutic antibodies and activate tumour‐specific T cells. (II) Using CRISPR/Cas9 editing technology to generate engineering organoids to mimic the genotypes and phenotypes of original tumours. (III) The bioluminescence system based on enhanced green fluorescent protein‐firefly luciferase transgenes allows for real‐time monitoring and quantifying the immune cell‐mediated killing effects. (IV) Using acoustic droplet printer in hydrophobic substrate to create many homogeneous tumour organoids, which undoubtedly modelled the immune microenvironment. (V) Micro‐CT images were used to quantitatively and visually analyse the distribution of macrophage‐like cells. (VI) The uniform immune organoids from acoustic cell assembly device integrated with sterile petri dishes are developed to preserve the phenotypes and viability of immune cells. (VII) Machine learning‐based image cytometry was used to estimate and quantify the immune parameters of organoids in co‐cultures. (VIII) OrganoIDNet algorithm was used to identify the therapeutic effects in organoids co‐cultured with PBMCs.

Although substantial progress has been made in the development of PDTOs for predicting anti‐tumour immune responses, there is a need for further optimisation of their yields and cultivation conditions. Currently, the experimental substrates, such as Matrigel and collagen, are common matrices for PDTO culture. They possess different characteristics and drawbacks. Matrigel, derived from Engelbreth–Holm–Swarm mouse sarcoma cells, comprises a mixture of laminin, collagen IV and other ECM proteins.^176^ It exhibits a gel‐like consistency and serves as a 3D scaffold for embedding and culturing organoid‐forming cells.^177^ Collagen matrices, highly abundant in the ECM of animal cells, mainly consist of collagen fibres.^178^ These matrices offer versatility in stiffness and pore size, depending on the concentration used, and can be employed alone or in combination with other factors for organoid culture.^179^ In addition, some specific functional biomaterials, particularly hydrogels (e.g., natural polymers and synthetic polymers), have presented promising prospects for improving the generation of physiologically relevant organoids.^180^, ^181^ Nevertheless, these materials exhibit evident drawbacks in organoid engineering and translational applications, including issues such as batch‐to‐batch variability and low reproducibility.^182^, ^183^ The exploration of novel biomaterials and synthetic technologies has the potential to pave the way for personalised immuno‐oncology therapies (Figure 5).

Currently, multiplex gene editing of organoids using lentiviral gene transduction or CRISPR/Cas9 has effectively replicated the genomic and phenotypic profiles of human cancers.^184^, ^185^ These genetically defined PDTOs have enabled rapid assessment of intricate TME and T cell‐dependent responses. A novel device integrating an acoustic cell assembly system with sterile Petri dishes has recently been developed to create organotypic cell clusters acoustically assembled within ECM hydrogels.^186^ This device allows hundreds of uniform clusters to be readily generated rapidly and consistently. More importantly, these cell clusters could preserve the phenotypes and viability of immune cells of the original TME.^187^ Gong et al.^188^ have employed an acoustic droplet printer on a hydrophobic substrate to establish another acoustic organotypic system successfully. This acoustic tool has emerged as a promising alternative for quickly generating many homogeneous bladder tumour organoids, effectively modelling all components within the immune microenvironment. In immunotherapeutic organoids, alginate‐poly‐l‐lysine microcapsules containing genetically modified cells have been used to secrete bispecific anti‐CEA/anti‐CD3 antibodies, thus activating tumour‐specific T cells.^189^ The bispecific antibody production system offers several notable advantages, including biosafety, cost effectiveness and the ability to generate therapeutic antibodies sustainably.^190^

Subsequently, the imaging‐based engineered platform have also been employed to improve the application values of organoids in pursuing human‐like immune systems.^191^ To bridge lung organoids with high‐resolution computed tomography (CT), Choi et al.^192^ obtained the micro‐CT images to quantitatively and visually analyse the distribution of macrophage‐like cells. Kholosy's group^159^ used a bioluminescence system based on enhanced green fluorescent protein‐firefly luciferase transgenes (eGFP‐ffLuc) to assess immunotherapy in paediatric neuroblastoma organoids visually. As expected, this optical system enables real‐time monitoring and quantification of immune cell‐mediated cytotoxic effects in response to anti‐GD2 monoclonal antibodies. Recently, Ferreira's colleague^193^ designed a deep‐learning‐empowered algorithm, OrganoIDNet, to identify the therapeutic effects in pancreatic cancer organoids co‐cultured with PBMCs. Live‐cell imaging in combination with OrganoIDNet could expectedly distinguish the organoid responses to PD‐L1 inhibitor over time. Similarly, another machine learning‐based image cytometry, integrated with end‐point assay and time‐lapse assay, was acquired to estimate and quantify the immune parameters of organoids in co‐cultures.^194^ These findings supported that the imaging assay is a straightforward methodology that allows for direct visualisation and observation of immunophenotypic features in immune‐tumour organoids.

These examples highlight the potential of emerging technologies to enhance the functional relevance of immune‐tumour organoids, offering valuable alternative methods to investigate communication between tumours and immune cells. Nevertheless, to thoroughly evaluate immunotherapy, refining how we can fully replicate the intricate immune microenvironment with long‐term reliability and robustness in organotypic models remains necessary. Further efforts are also essential to expand the utility of modified immunocompetent organoids to support clinical decision‐making. However, translating immune organoid into clinical practice holds some outstanding questions, such as experimental standardisation, long‐term cultivation and accurately maintaining cellular complexity.^127^, ^195^ Repeatable and standard generation protocols for immune organoids should be specified to minimise the inconformity from different laboratories or hospitals.^196^ Beyond identifying the underlying molecular mechanisms of resistance, immune organoids have the potential to improve therapeutic endeavours, such as optimising existing immune‐regulatory strategies and accessing novel treatment methods. Moreover, organoids offer the possibility to real‐time determine the clinical responses to immunotherapy. After addressing these challenges, emerging methodologies are expected to significantly drive the clinical translation of immune organoids, bringing us closer to realising the substantial promise of immune oncology interactions.

In terms of potential for clinical applications, PDTOs could accurately replicate the 3D architecture and therapeutic response of tumours, highlighting their distinguished potency for bridging bench‐to‐bedside applications. Multiple reports have demonstrated the application of PDTOs for response evaluation of cancer treatment, including chemotherapy and targeted agents. Patient‐derived colorectal cancer organoids function as practical and effective in vitro tools for investigating the tumour response to EGFR‐targeted therapy.^197^ The interaction between CD44 receptor and hyaluronan upregulated the expression of drug efflux transporters and driven chemoresistance in pancreatic ductal adenocarcinoma organoids.^198^ However, drug response could be frequently dependent on the interactions among components in TME. Traditional organoids that consist only of tumour cells fail to replicate the complex TME, limiting their ability to accurately mimic therapeutic responses.^199^ Conversely, the models incorporating stromal and immune elements might augment the clinical translational potential of organoids. Moreover, it is anticipated that the utilisation of immune organoid models to define the response to cancer treatment could ideally be performed rapidly and reliably. Patient‐derived micro‐organospheres, which maintain the infiltration of stromal cells and immune cells, providing a clinical model for assessing the therapeutics in immuno‐oncology. The micro‐organosphere systems could also be used to guide the timely clinical decision‐making for cancer immuno‐oncology therapies.^81^ The multicentre observational trials to estimate therapy outcomes and personalised medicine are currently performed in immune organoids of head and neck squamous cell carcinoma.^200^ Taken together, these findings provide meaningful technologies for mimicking the full diversity of immune and non‐immune components, thereby enhancing the in vitro modelling of therapies in cancer patients.

## 10 CONCLUSION

Immunotherapeutic strategies have emerged as groundbreaking technologies in cancer treatment, offering promising approaches to control cancer cells. Consequently, there is growing interest in using patient‐derived biomaterials to explore dynamic interactions between the tumour and the immune system, significantly impacting the effectiveness of individualised immunotherapy. PDTOs, 3D culture systems derived from tumour tissues of patients, have the potential to capture the heterogeneity, genotypes and phenotypes of original tumours, predict therapeutic responses and guide personalised therapies. In this review, we have summarised the characteristics and functions of PDTOs and emphasised their applications in anti‐tumour immunological responses. Although several limitations remain to be addressed, established organotypic models positively impact assessing responses to immunotherapy. Using human organoid culture to model the immune microenvironment will ultimately open comprehensive and effective therapeutic avenues to eliminate cancer cells.

## AUTHOR CONTRIBUTIONS

D. C., L. X.. and Q. C. wrote this review article. M. X. performed technical and administrative support. C. X. designed the review and contributed to manuscript preparation. All authors reviewed and approved the final version of the manuscript.

## CONFLICT OF INTEREST STATEMENT

The authors declare that there are no conflict of interest.

## FUNDING INFORMATION

This work was supported by the China Postdoctoral Science Foundation (2023M743200) and the Henan Medical Science and Technology Joint Building Program (LHGJ20230239 and LHGJ20230160).

## ETHICS STATEMENT AND CONSENT TO PARTICIPATE

Not applicable.

## CONSENT FOR PUBLICATION

Not applicable.

## Data Availability

The data used to support this review are included within the article.
